# Translator's Style Through Lexical Bundles: A Corpus-Driven Analysis of Two English Translations of Hongloumeng

**DOI:** 10.3389/fpsyg.2021.633422

**Published:** 2021-05-28

**Authors:** Kanglong Liu, Muhammad Afzaal

**Affiliations:** ^1^Department of Chinese and Bilingual Studies, The Hong Kong Polytechnic University, Kowloon, Hong Kong; ^2^Institute of Corpus Studies and Applications, Shanghai International Studies University, Shanghai, China

**Keywords:** translator's style, lexical bundles, hongloumeng, corpus-assisted translation studies, functional classification

## Abstract

Based on a corpus-driven analysis of two translated versions of *Hongloumeng* (one by David Hawkes and the other by Xianyi Yang and Gladys Yang) in parallel corpora, this article investigates the use of lexical bundles in an attempt to trace the stylistic features and differences in the translations produced by the respective translators. The *Hongloumeng* corpus is developed at the sentence level to facilitate co-occurrence of the source texts and the two corresponding translations. For this purpose, the three-word and four-word lexical bundles were first extracted and then analyzed with respect to the functional classification proposed by Biber et al. (2004). The results of the study show that Hawkes' translation is embedded with a greater number and variety of lexical bundles than the one by the Yang couple. The study also identified the differences between the two versions which can be traced back to the deployment of different translation strategies of the translators, appearing in turn to be influenced by the language backgrounds of the translators, the translation skopos and settings, and the social, political, and ideological milieu in which the translations were produced.

## Introduction

*Hongloumeng* (《紅樓夢》), also known as *The Story of the Stone*(*Shitouji* 《石頭記》), has long been acclaimed as one of the greatest masterpieces of Chinese literature because of its kaleidoscopic depiction of almost every aspect of Chinese culture. The 120-chapter novel is said to have been written by two authors. The first 80 chapters are authored by Xueqin Cao (Hsueh-ch'in Ts'ao) and the remaining 40 chapters are believed to have been written by Gao E in the Qing Dynasty in China (1644–1911). The various themes covered by the novel, including a love tragedy, Daoist–Buddhist enlightenment, social observation, the decay of an aristocratic family, and even a veiled attack on Manchu rule, have made it a pearl of Chinese literature. It has been translated into almost all the major languages in the world because of its literary status in China. It is perhaps the most well-researched literary work in China. There are journals specifically devoted to studies of the many aspects of *Hongloumeng*, ranging from linguistic and religious to sociological perspectives due to the profound cultural connotations and ideological contents of the novel. In the translation field, researchers have long been interested in comparing the translation strategies employed by the different translators working on the same text. As *Hongloumeng* depicts so many aspects of Chinese cultural life, the translations, to a certain extent, reflects the many core problems frequently debated in the field of translation studies. These include questions over whether translators should adopt a domesticating or foreignizing approach in rendering the culture-specific items in the novel, strike a balance between readability and the source-text cultural elements or empoly an appropriate translation technique in translating this classic literary work.

In recent years, corpus-based techniques have been fruitfully applied to the investigation of translated literary works like *Hongloumeng* to examine the translational features of the texts. Baker in a number of her research 1993; 1995; 1996; 2004 has greatly advanced the corpus-based methodology to examine the linguistic features of translational language. Following Frawley (1984) that translational language is considered the “third code” distinctive from both source and target languages, Baker proposed the well-known translation universals (TUs) hypotheses that are concerned with the unique features of translational language for corpus-based investigations. Since then, there has been an increase in the number of corpus-based studies and many were carried out with the aim of testing the TU hypotheses. The TUs examined include simplification (Malmkjær, 1997; Laviosa, 1998), explicitation (Øverås, 1998; Olohan and Baker, 2000; Olohan, 2003), normalization (Kenny, 2001, 2017), leveling out (Laviosa-Braithwaite, 1996), and unique item hypothesis (Eskola, 2004; Tirkkonen-Condit, 2004).

Despite the dearth of research on TU, the universality of translated texts has been controversial and increasingly challenged by recent corpus-based research. For example, researchers have found that other variables such as source language (Williams, 2005; Ferraresi et al., 2018) and genre (Kruger and Van Rooy, 2012) can affect the profiling of translated texts besides translation status. As different translators speak different first languages, come from different backgrounds, receive different professional training, and serve different translation purposes, stylistic variance, which can distinguish a translator's style, unavoidably exists. In comparison with the controversies surrounding the concept of TUs, corpus-assisted translation style research using similar measures is relatively less disputable and has been fruitfully pursued in the field of translation studies. Shrefler (2010) and Mastropierro (2018) are among the scanty studies, which examined and explained how quantifiable features such as lexical bundles can help us better describe and better understand a translator's style. Using the functional classification proposed by Biber et al. (2004), Shrefler (2010) adopted a corpus-based approach to compare two German translations, at different times, of Bible, with the aim to test the correlation between readability and the density of lexical bundles. He found that Luther's version used more organization markers, rendering reading easier for people at that time; at the same time, the lack of referential bundles may also hinder reading. Lexical bundles is an important parameter for studying translator's style in other studies as well, for example, Lee (2013) also applied the same functional classification and found that Korean news translators intentionally limited the use of hedging bundles, which are conventional in Korean news language. This is a special phenomenon exclusive to Korean news translators. When comparing professional and student translators' works, Novita and Kwary (2018) found that professional translators used more types of lexical bundles and more frequently; they further concluded that the use of lexical bundles is correlated to the translator's proficiency of the target language, which is in line with Paquot's (2013) findings that non-native English speakers are likely influenced by their first language (L1 transfer) and use lexical bundles differently.

The studies discussed earlier show that quantifying stylistic features like lexical bundles can shed light on translator's styles. Traditionally, researchers have tended to adopt a phraseological approach to investigating the structure and functions of multiword language units with the aim of using linguistic labels and criteria to describe formulaic language (e.g., Cowie, 1998). Apart from “lexical bundles” (Biber and Conrad, 1999; Biber et al., 2003, 2004), a number of terms have been used to refer to the recurrent strings of a pre-established number of words, such as “formulaic sequences” (Simpson-Vlach and Ellis, 2010), clusters (Mastropierro, 2018), multi-word units (Mitkov et al., 2018), and “n-grams” (Mahlberg, 2013, p. 48). The corpus-based methods take a completely different approach by calculating the frequency of the word strings in a corpus, a feature that was previously overlooked but prevalent in natural language. As demonstrated by the recent studies mentioned above, we can see that by counting the frequency and categorizing the functions of lexical bundles one can draw insights of translators' idiosyncratic style (e.g. over use of organization markers). Despite the plentiful *Hongloumeng* translation research produced every year, many fail to discuss the subtle difference between David Hawkes' and Yang couple's translation versions with quantifiable empicial evidence. As comparing the two translations without quantifiable evidence is apparently subjective and opinionated, we argue that at least the frequencies, if not more, of the stylistic features in question should be compared with lend stronger evidence to analyze literary translations, especially of monumental literary masterpieces like *Hongloumeng*. This article demonstrates how to use corpus linguistic methods to benefit and assure the quality of literary translation studies in a scientific manner. Lexical bundles were taken as an entry point to probe into the different translation styles of the two *Hongloumeng* translators since lexical bundles have yielded fruitful findings in other translation style research. The main goal of the current study was to investigate the “translator's style” (Baker, 2000) in two English versions of *Hongloumeng*, by addressing the following two research questions: (1) How are lexical bundles differently represented in the two English translations of *Hongloumeng*? (2) What are the possible factors that lead to such differences between the two translation versions of *Hongloumeng*?

## Methodology

The concept of “translator's style” put forward by Baker has a direct connection with corpus-based translation studies. Huang and Chu (2014) conducted a detailed review on the translator's style and proposed that there are two models in this area of research, namely the monolingual comparable corpus (e.g., Laviosa, 1998; Olohan and Baker, 2000; Olohan, 2003; Chen, 2006) and the parallel model (e.g., Øverås, 1998; Kenny, 2001), with the first one looking at textual features of translations without the same Source Text (ST) and the latter with the same ST. The methodology proposed by Baker (2000) in which she selected the English translations of two translators from the Translated English Corpus (TEC) for investigating the distinctive features of the respective translator is deemed to be one of the comparable models. The method proposed by Baker (2000) has been adopted in a number of studies to investigate distinctive features of translator style. Such research ranges from investigating the use of contracted forms by two different translators (Olohan, 2003) to inquiring into the stylistic differences in the use of foreign words in translations (Saldanha, 2011). In all these studies, the translations were analyzed and compared without direct reference to the ST.

The comparison of target texts (TTs) without reference to the same ST can be problematic because the stylistic differences cannot be directly attributed to the translator(s) due to the possibility of the influence of multiple styles of ST-related elements. Hence, Mastropierro (2018, p. 242) has strongly argued for the need to make use of the parallel model in investigating the translator's style because such a model “would facilitate the process of attributing stylistic features to the translator alone.” Applying the parallel model in which the ST was taken as one variable for analysis and comparison, Bosseaux (2004, 2007) specifically looked into indirect speech within Virginia Woolf's novels and their corresponding French translations. Her main aim was to uncover “the nature of the translator's discursive presence by exploring certain narratological aspects of the relation between the originals and translations” (Bosseaux, 2004, p. 107), including deixis, modality, transitivity, and free indirect discourse. Winters (2004a,b, 2007, 2009) also made use of a similar methodology to examine differences in translator's style by inquiring into the German translations of F. S. Fitzgerald's *The Beauty and Damned*. This entailed comparing the distinctive use of modal particles, loan words, code switches and speech-act report verbs in the translations of the novel produced by two different translators. The results show that the two translations differ to a great extent in all these aspects. Therefore, the current study was based on the parallel model. The aim of the study was to identify and compare the use of lexical bundles in the two English translations of *Hongloumeng*. In line with the idea that “source–target relationships and questions of similarity and difference are intrinsic to the investigation of translational phenomena” (Halverson, 2007, p. 109), this study took the view that the ST, among a number of possible factors, exerted a particularly significant influence on the TT. Thus, the parallel model was viewed as being clearly advantageous for studying the stylistic differences in translations produced by different translators.

The corpus for the study comprises the first 15 chapters of *Hongloumeng* (in Chinese) published by People's Literature Press and two corresponding English translations, one by David Hawkes (hereinafter “Hawkes”) and the other by the Yang spouses (hereinafter “Yangs”). The corpus has been aligned at the sentence level for facilitating co-occurrence of the ST and its two corresponding translations. The Chinese portion of the corpus contains 91,173 Chinese characters, with the Hawkes translation comprising 89,396 running words and the Yangs translation consisting of a total of 67,649 running words plus 761 words of footnotes. The study used a corpus tool Wordsmith 6.0 (Scott, 2012) to retrieve the lexical bundles. The three-word, four-word, five-word and six-word lexical bundles were searched and retrieved using the Wordlist function of Wordsmith. The lexical bundles were then cross-tabulated, categorized, and compared using Microsoft Excel. It should be noted that the shorter lexical bundles are often contained in larger ones. For instance in Tables 1, 2, the six-word bundles taken from Hawkes and Yangs show that the small bundles are part of the larger bundles. As prefabricated chunks stored in the memory, these bundles work as building blocks for the formation of the larger ones.

**Table 1:** Example of expanding lexical bundle in Hawkes.

**Length**						
3	you	will	have			
4	you	will	have	to		
5	you	will	have	to	read	
6	you	will	have	to	read	the

**Table 2:** Example of expanding lexical bundle in Yangs.

**Length**						
3	on	the	edge			
4	on	the	edge	of		
5	on	the	edge	of	the	
6	on	the	edge	of	the	kang

Biber et al. (1999) pinpointed that three-word lexical bundles are “extremely common” collocational patterns whereas four-word bundles up are more phrasal and uncommon in nature. This is confirmed by our preliminary analysis: in comparison to three-word bundles, four-word bundles were found to occur to a relatively lesser extent but nonetheless provided an entry point for a more detailed analysis of unique bundle use in the two translation versions; four-word clusters also appeared to be preferred over the five-word bundles as the latter occurred far less frequently in the texts, thus posing the potential problem of underuse. Since the current study aims to study and compare the lexical bundles as a parameter to pinpoint different translators' style, we are interested in the signature four-word bundles indeed. Hence, this paper mainly investigated the lexical bundles by systematically studying the four-word lexical bundles in both translations.

This study focused on lexical bundles that appeared at least five times in either of the English texts. In addition to a comparative study of the number of lexical bundles at each length, the decreased rate of the number of lexical bundles as a result of the length increase was also investigated. Therefore, the top 20 four-word lexical bundles of each English translation were categorized using this taxonomy before the frequency levels off after seven (as mentioned, the minimum frequency of lexical bundles were set at five occurrences). A special focus was also accorded to examining the nature of these bundles and their functions in the text. The lexical bundles were categorized with reference to the functional classification of four-word lexical bundles proposed by Biber et al. (2004). The framework includes “stance expressions” which express epistemic or attitudinal/modal stances, “discourse organizers” which help introduce/focus or clarify/elaborate topics, “referential expressions” which identify focus and imprecision or specify attributes and time, and “special conversational functions.” A qualitative study of the functional variety and nature of these lexical bundles was then conducted to probe into the salient features of the translated texts.

## Results

Based on the comparison of lexical bundles, the quantitative results of the two *Hongloumeng* translations have been presented in this section. Table 3 shows the number of lexical bundles of varying lengths. First, the data suggest that as the number of lexical bundles found in the texts decrease, the bundle length increases. Second, in comparing the incidence of lexical bundles in Hawkes and Yangs, it is found that the Hawkes translation contains more types and tokens of lexical bundles than Yangs in all bundle lengths. Third, except for the six-word lexical bundles, the decrease rate of the number of types and tokens as length increases is greater on average in Yangs than in Hawkes. For example, while the number of the types and tokens of four-word lexical bundles of Hawkes is 19.6 and 17.2% of the previous length, they are relatively lower in Yangs (18 and 15.7%, respectively). The analysis also shows that the lexical bundles account for a much greater proportion of the text in Hawkes than they do in Yangs. For example, the three-word bundles, with a total of 10,346 words, account for 11.6% of the text in Hawkes. In contrast, the Yangs features only 1,331 occurrences of three-word lexical bundles with a total of 5,914 words, thus accounting for only 8.7% of the corpus.

**Table 3:** Statistical comparison of lexical bundles.

**Number of words**	**Hawkes**		**Yangs**	
	**Types**	**Tokens**	**Types**	**Tokens**
Three	2,192	10,346	1,331	5,914
Four	429	1,781	240	931
Five	100	390	54	196
Six	20	78	18	59

Tables 4, 5 present the top 20 most frequent four-word lexical bundles found in the translations by Hawkes and Yangs. However, the contractions are treated as two words. For example, “don't” is a contraction that is short for “do not,” the apostrophe in “don't” takes the place of the missing “o,” and in the current study “don't” is treated as two words, namely “do not.”

**Table 4:** Top 20 most frequent four-word lexical bundles in Hawkes.

**N**	**Freq**.	**Four-word lexical bundles**				**Function**	**Category**
1	67	zhou	rui	s	wife	Referential	Person
2	14	i	don	t	know	Stance expressions	Topic introduction
3	13	the	rong	guo	mansion	Referential	Place
4	12	members	of	the	clan	Referential	Person
5	12	the	ning	guo	mansion	Referential	Place
6	11	of	the	ning	guo	Referential	Place
7	11	said	zhou	rui	s	N/A	Person
8	9	at	the	same	time	Discourse organizers	Time
9	9	if	you	don	t	Stance expressions	attitudinal/modality stance
10	9	in	the	midst	of	Referential	Intangible framing attribute
11	8	in	the	course	of	Referential	Intangible framing attribute
12	8	in	the	form	of	Referential	Tangible framing attribute
13	8	it	would	be	a	stance Expressions	Attitudinal/modality stance
14	8	lady	xing	and	lady	Referential	Person
15	8	the	edge	of	the	Referential	Tangible framing attribute
16	8	xing	and	lady	wang	Referential	Person
17	7	as	soon	as	they	discourse organizers	time
18	7	bao	yu	and	qin	Referential	Person
19	7	by	the	hand	and	Referential	Body part
20	7	don	t	know	what	Stance expressions	Attitudinal/modality stance

**Table 5:** Top 20 most frequent four-word lexical bundles in Yangs.

**N**	**Freq**.	**Four-word lexical bundles**				**Function**	**Category**
1	14	the	lady	dowager	s	Referential	Person
2	10	to	the	lady	dowager	Referential	Person
3	9	of	the	jung	mansion	Referential	Place
4	8	hsing	and	lady	wang	referential	Person
5	8	if	you	don	t	Stance expressions	Attitudinal/modality stance
6	8	lady	hsing	and	lady	Referential	Person
7	8	on	the	edge	of	Referential	Tangible framing attribute
8	8	pao	yu	and	chin	Referential	Person
9	8	the	duke	of	ningkuo	Referential	Person
10	8	yu	and	chin	chung	Referential	Person
11	7	chia	sheh	and	chia	Referential	Person
12	7	garden	of	concentrated	fragrance	Referential	Place
13	7	in	the	jung	mansion	Referential	Place
14	7	in	the	ning	mansion	Referential	Place
15	7	of	the	duke	of	Referential	Person
16	7	the	edge	of	the	Referential	Tangible framing attribute
17	7	the	garden	of	concentrated	Referential	Place
18	7	the	two	of	them	Referential	Person
19	7	there	s	no	need	Stance expressions	Attitudinal/modality stance
20	7	to	pay	his	respects	N/A	N/A

### Lexical Bundles in Patterns

As Tables 4, 5 show, in both translations, referential expressions account for more than half of the four-word bundles retrieved. They are used to refer to characters and places of the novel and a variety of entities. This is particularly the case in Yangs. In Hawkes, the top 20 four-word lexical bundles present a variety of structural patterns. The majority of the bundles are referential expressions, but among the 13 referential expressions, six refer to people, six comprise place names, two indicate tangible framing attributes (Nos. 12 and 15) and two refer to intangible framing attributes (Nos. 10 and 11). The rest of the seven four-word bundles are either discourse organizers or stance expressions. Thus, in addition to people and place bundles, there are still 11 other bundles in Hawkes, thus suggesting the use of a greater bundle variety than in Yangs. On the contrary, Table 5 shows that the most of the bundles in Yangs are referential in nature. There are 17 referential expressions and two stance expressions (“if you don't” and “there's no need”). One bundle (No. 7) cannot fit into the functional categories proposed by Biber et al. (2004). However, these bundles are predominantly people (10 instances) and place names (five instances). The other two bundles (Nos. 7 and 16) are actually part of the five-word bundle “on the edge of the.” It can be seen here that discourse organizers in Yangs are not used to the same extent as in Hawkes. Clearly, Hawkes has a greater number and variety of bundles than Yangs.

### Lexical Bundles in Context

As stated in the above section, the majority of lexical bundles in Yangs comprise people and place names, which are story-exclusive and comparatively less relevant to study translator's style. In view of this, these people and place bundles were excluded in the qualitative analysis. With the exclusion of these bundles, 11 other bundles are left in Hawkes and four remain in Yangs. The investigation of the contextual use of the bundles was based on the 11 bundles found in Hawkes, with special focus on comparing the extent to which Hawkes diverges from Yangs in the use of these bundles in actual translations. Due to the vast number of translation examples, only certain typical excerpts were selected for scrutiny.

#### Hawkes: I don't know

The lexical bundle “I don't know” occurs 14 times in Hawkes and only six times in Yangs. Apparently, Hawkes is more inclined to render the text using this bundle than Yangs. According to Aijmer (p. 158), “*I don't know* occurs in an interactionally and pragmatically interesting position where it marks a stepping-stone to what comes next, signaling the starting-point of a speaker perspective.” She further points out:

*I don't know* can, for instance, have the polite function of conveying deference or of softening an assertion or it can have mainly a speech management function to facilitate production and processing by adding to the coherence of the discourse (2009, p. 157).

In Example (1), the Yangs uses “don't think you can fool me” while Hawkes uses more words to convey the same idea by adding “do you think” before “I don't know” and the sentence seems more colloquial and conversation-like. This is also the case with Example (2) where Hawkes uses “I don't know” while the Yangs uses “Do tell me” for translation. In these two examples, we can also see a difference in terms of the perspective of interpretation.

(1) ST: 每日家偷狗, 爬灰的爬灰, 小叔子的小叔子, 我什不知道？

Hawkes: Up to their dirty little tricks every day. I know. Father-in-law pokes in the ashes. Auntie has it off with nervy. Do you think I don't know what you're all up to?

Yangs: Little did he expect to beget such degenerates, a houseful of rutting digs and bitches in heat, day in and day out scratching in the ashes and carrying on with younger brother-in-law. Don't think you can fool me.

(2) ST:玉是一仙姑, 喜的忙作揖道: ”神仙姐姐不知那, 如今要往那去？

Hawkes: Observing delightedly that the lady was a fairy, Bao-yu hurried forward and saluted her with a smile. “Madam fairy, I don't know where you have come from or where you are going to.”

Yangs: Overjoyed by the apparition of this fairy, Pao-yu made haste to greet her with a bow.” Sister Fairy.” He begged with a smile. “Do tell me where you are from and whither you are going.”

#### Hawkes: At the Same Time

The lexical bundle “at the same time” occurs nine times in Hawkes, but not found in Yangs. Halliday and Hasan (1976, p. 242–243) lists this item as an example of a temporal conjunction for specifying conjunctive relations. This is the case in Example (3). It is evident that the rendering in Hawkes is more colloquial and closer to daily speech while the Yangs' version sounds more “written” than “spoken.” On the other hand, Nesi and Basturkemen (2009, p. 34–35) states that although its residual meaning suggests a temporal relationship, “at the same time” can also signal contrast. This is evident in Example (4), wherein Hawkes uses the bundle “at the same time” with the antecedent “yet” to convey a fluctuating succession of information. In Yangs, the rendering is rather straightforward as reflected by the use of the conjunction “and,” which makes the translation appear rather plain when compared with the translation in Hawkes.

(3) ST: 熙了, 忙悲喜道: ”正是呢！我一了妹妹, 一心都在他身上了, 又是喜, 又是心, ，竟忘了老祖宗。

Hawkes: In obedience to the command Xi-feng at once exchanged her grief for merriment. ‘Yes, of course. It was just that seeing my little cousin here put everything else out of my mind. It made me want to laugh and cry all at the same time. I'm afraid I quite forgot about you, Grannie dear.

Yangs: Hsi-feng switched at once from grief to merriment. “Of course,” she cried. “I was so carried away by joy and sorrow at sight of my little cousin, I forgot our Old Ancestress.

(4) ST: 那玉合上眼, 便惚惚的睡去, 似秦氏在前, 遂悠悠, 了秦氏, 至一所在。

Hawkes: As soon as Bao-yu closed his eyes he sank into a confused sleep in which Qin-shi was still there yet at the same time seemed to be drifting along weightlessly in front of him.

Yangs: Pao-yu fell asleep as soon as he closed his eyes and dreamed that Ko-ching was before him.

#### Hawkes: If You Don't

The lexical bundle “if you don't” functions as a stance expression according to the categorization offered by Biber et al. (2004). It is used mainly for topic introduction or focus. As can be seen in Example (5) from the Hawkes' version, “if you don't” functions as a speech–act conditional in which the performance “represented in the apodosis is conditional on the fulfillment of the state described in the protasis” (Sweetser, 1990, p. 118). In most cases, the if-clause speech–act conditionals are addressee-oriented by nature. Based on the examples taken from Hawkes, we can clearly see that Hawkes tends to adopt a second-person perspective, while the Yangs are inclined to use first-person perspective in translating the dialogues in this novel. Also, Yangs' renderings are relatively more direct and plainer, thus presenting a different locutionary force than the translations made by Hawkes. This is also the case in Example (6), wherein Hawkes uses “if you don't mind… I shall…” and the Yangs simply translate it into “do you mind….” Again, the use of speech-act conditionals shows that Hawkes is more varied in terms of pragmatic functions.

(5) ST: 不和我的可, 若再的, 咱刀子去白刀子出!

Hawkes: Well, I'll tell you something. You'd better watch out. Because if you don't, you're going to get a shiny white knife inside you, and it's going to come out red!

Yangs: “Shut up, and I'll overlook it. Say one word more, and I'll bury a white blade in you and pull it out red!”

(6) ST: 士慌的忙起身罪道: ”恕之罪, 略坐, 弟即陪。

Hawkes: Shi-yin hurriedly rose up and excused himself: “I seem to have brought you here under false pretenses. I do hope you will forgive me. If you don't mind sitting on your own here for a moment, I shall be with you directly.”

Yangs: Then Shin-yin excused himself, saying. “Forgive my rudeness. Do you mind waiting here for a few minutes?”

#### Hawkes: In the Midst of; in the Course of; in the Form of

These three lexical bundles are all referential in nature and they are categorized as framing attributes. The two items “in the midst of” and “in the course of” are rather similar in meaning and they are both intangible framing attributes; “in the form of” is a tangible framing attribute. The two intangible attributes are not found in Yangs^1^ while there are six occurrences of “in the form of” in Yangs (as compared with eight occurrences in the Hawkes). Intangible framing attributes tend to be quite common in English academic prose (Biber et al., 2004; Biber, 2006). This difference is pertinent given that Hawkes and the Yangs belong to different cultural backgrounds. In Example (7), Hawkes uses “in the midst of discussing” while Yangs simply uses the present participle of a verb “discussing.” In Example (8), “in the course of” is used by Hawkes while the Yangs simply use “during” to convey a similar idea. The tangible framing attribute “in the form of” is used in both Hawkes and Yangs, yet it appears twice as frequently in the former than in the latter. In Example (9), Hawkes uses a verb (“manifest”) preceding the lexical bundle “in the form of” while the Yangs merely use the verb “transform.” Clearly, the use of lexical bundles is more prevalent in Hawkes than in Yangs.

(7) ST: 黛玉同姊妹至王夫人, 王夫人兄嫂的使家, 又姨母家姨母家遭人命官司等。

Hawkes: When Dai-yu and the girls went to call on lady Wang, they found her in the midst of discussing family affairs with the messengers from her elder brother and his wife and heard talk of their aunt's family in Nanking being involved in a case of manslaughter.

Yangs: To resume, Tai-yu and other girls found Lady Wang discussing family affairs with messengers sent by her brother, and heard that her sister's son was involved in a murder case.

(8) ST: 金氏了半日, 把方才在他嫂子家的那一要向秦氏理的盛, 早的都在爪去了。

Hawkes: Mrs. Huang's determination to have things out with Qin-shi, of which she had boasted so valiantly at her sister-in-law's, had, in the course of this outpouring, fled to the far kingdom of java.

Yangs: Aunt Huang's furious determination while with her sister-in-law to have it out with Ko-ching had, during this recital, been scared away into the Sea of Java.

(9) ST: 所之秀, 漫所, 遂甘露, 和, 洽然溉及四海。

Hawkes: Moreover, an unused surplus of this pure, quintessential humor, unable to find corporeal lodgement, circulates freely abroad until it manifests itself in the form of sweet dews and balmy winds, aspersed, and effused for the enrichment and refreshment of all terrestrial Life.

Yangs: The over-abundance of this good essence, having nowhere to go, is transformed into sweet dew and gentle breezes and scattered throughout the Four Seas.

#### Hawkes: It Would Be a

The bundle “it would be a” expresses a conditional situation which is hypothetical in nature. The modal verb “would” can be used to talk about a doubtful future situation even if it is not known to be counterfactual. By comparing the two translations in Example (10), we can see that Hawkes' translation is softer in tone than the one by the Yangs, with the latter using the modal verb “will.” In comparison to “would,” “will” is typically used to talk about simple futurity which expresses the most certainty. In Example (11), Hawkes uses “it would be a journey wasted” while Yangs renders it as “it's no use my going.” Again, Hawkes' version is a conditional statement while the one in Yangs is more declarative and direct. This confirms the pervious findings that Hawkes is more varied in terms of pragmatic functions.

(10) ST: 今依傍外祖母及舅氏姊妹去, 正好我盼之, 何反不往?

Hawkes: It would be a great weight off my mind to know that you had your grandmother Jia and your uncles' girls to fall back on. I really think you ought to go.

Yangs: If you go to stay with your grandmother and uncles' girls, that will take a great load off my mind. How can you refuse?

(11) ST: 姥姥道: ”!可是的, ‘侯深似海’, 我是什西, 他家人又不得我, 我去了也是白去的。

Hawkes: “Bless us and save us!” said Grannie Liu. “You know what they say: ‘a prince's door is like the deep sea.’ What sort of creature do you take me for? The servants there don't know me; it would be a journey wasted.”

Yangs: Aiya! The threshold of a noble house is deeper than the sea. And who am I? The servants there don't know me. It's no use my going.

#### Hawkes: the Edge of the

The lexical bundle “the edge of the” occurs eight times in Hawkes and seven times in Yangs. It seems that Hawkes and the Yangs adopt a similar approach when using this tangible framing attribute. In fact, this is one of the few bundles with a similar distribution in both Hawkes and Yangs. In most cases, it co-occurs with the noun “kang” (a traditional long sleeping platform made of bricks or other forms of fired clay). The novel is replete with conversations taking place besides the “kang.” As far as the number of occurrences is concerned, it might be that the descriptions of tangible locations are likely to be similar despite the translators' cultural differences.

(12) ST: 姥姥已在炕沿上坐了。

Hawkes: …and Grannie Liu sat herself down on the edge of the kang.

Yangs: By now Granny Liu had seated herself on the edge of the kang,

(13) ST: 黛玉站在炕沿上道: ”唆什, ,我瞧瞧。

Hawkes: “Come here!” said Dai-yu standing on the edge of the Kang. “I'll put it on for you!”

Yangs: “What a commotion!” Tai-yu stood up on the kang. “Come here. Let me see to it.”

#### Hawkes: As Soon as They

As a discourse organizer, “as soon as they” occurs seven times in Hawkes, but it is not found in Yangs^2^. However, the three-word bundle “as soon as” does appear in both texts. It appears 34 times in Hawkes and 18 times in Yangs, with the occurrence of the bundle being twice as frequent in the former than in the latter. As a non-idiomatic phrase, “as soon as” can be used to describe two actions or events that happen very quickly one after the other. The immediacy of the event is better described in Hawkes than in Yangs, as is evident in the two examples (Example 14 and 15) provided below:

(14) ST: 一姐入茅堂, 因命玉等先出去。

Hawkes: As soon as they were inside the thatched central building, Xi-feng asked the boys to amuse themselves outside.

Yangs: Once in the thatched house Hsi-feng suggested to Pao-yu that he should amuse himself outside.

(15) ST: 晴雯道: ”快提。一送了, 我知道是我的, 偏我才吃了, 就放在那。

Hawkes: “Don't talk to me about those dumplings!,” said Skybright. “As soon as they arrived I realized that they must be for me, but as I'd only just finished eating, I put them on one side meaning to eat them later.”

Yangs: “Don't ask!” answered Ching-wen. “I knew at once they were meant for me, but as I'd just finished my breakfast I left them here.”

#### Hawkes: by the Hand and

The lexical bundle “by the hand and” occurs seven times in Hawkes, but it is not found in Yangs^3^. As a referential bundle, “by the hand” always appears in the verb phrase “take somebody by the hand.” In Example (16), “攜手” (literally “take hand”) is retained and translated in Hawkes, while this vivid description is omitted in Yangs. In Example (17), the difference is also noticeable when comparing Hawke's version of “Xi-feng took Dai-yu by the hand” and Yangs' version of “Hsi-feng took her hand.” It can be seen that the two translations differ in the expression of a possessed body part: in the case of Hawkes, the possessor (Dai-yu) is treated as the object of the verb (took), with the possessed body part expressed in a prepositional phrase (by the hand). On the other hand, in the case of Yangs, the possessed body part is treated as the direct object (her hand) of the verb (took), which is the usual expression in the Chinese language. Based on these two examples, we can see that Yangs' renditions are syntactically closer to the Chinese ST as they evidence the adoption of a word-for-word translation strategy.

(16) ST: 著, 便令人送女去, 自雨村手至房中。小童茶。

Hawkes: So saying, he called for a servant to take the child indoors, while he himself took Yu-cun by the hand and led him into his study, where his boy served them both with tea.

Yangs: He told a servant to take his daughter inside, and led Yu-tsun into his study, where a boy served tea.

(17) ST: 熙著黛玉的手, 上下打了一回, 仍送至母身坐下。

Hawkes: Xi-feng took Dai-yu by the hand and for a few moments scrutinized her carefully from top to toe before conducting her back to her seat beside grandmother Jia.

Yangs: Hsi-feng took her hand and carefully inspected her from head to foot, then led her back to her seat by the Lady Dowager.

#### Hawkes: don't Know What

The lexical bundle “don't know what” occurs seven times in Hawkes, but it is not found in Yangs^4^. In Hawkes, “don't know what” is part of the five-word bundle “I don't know what” which occurs five times in the corpus. According to Biber et al. (1999, p. 989), “I don't know what” is one of the common lexical bundles found to occur in conversation. If we interpret along this line, we can arrive at the conclusion that Hawkes' translations are more colloquial and in a sense more conversation-like, as shown by Examples (18) and (19) below:

(18) ST: 姐道: ”不知什原故。

Hawkes: ‘I don't know what the reason can be,’ said Xi-feng.

Yangs: “I wouldn't know,” Hsi-feng replied.

(19) ST: 秦笑道: ”可是有的。

Hawkes: ‘I don't know what you're talking about.’

Yangs: “You're just making that up!” protested Chin Chung.

## Discussion

Based on the data and analyses described above, some general trends can be observed: (1) Hawkes utilizes many more lexical bundles than Yangs which uses fewer bundles in both frequency and variety, and (2) Yangs adopts a more literal approach in translating the Chinese ST, whereas (3) it is clearly evident that Hawkes' adoption of lexical bundles has imbued his translation with a wider range of stylistic variations. In Hawkes, the stance expression bundles such as *I don't know* and *don't know what* help construe a conversational tone; *if you don't* which involves second personal pronoun sounds more addressee-oriented; the referential bundles *in the midst/course/form of* and *by the hand of* bring the focus to some particular attribute of the entity/reality; the discourse organizer bundles *at the same time* and *as soon as they* help strengthen the relationship between prior and coming discourse. Yangs has, on the other hand, adopted a smaller range of lexical bundles due to his relatively literal translation approach aiming to be faithful to the source text.

There is a significant difference in the use of lexical bundles in terms of both frequency and variety between Hawkes and the Yangs (see Tables 3–5). In many cases, high frequency lexical bundles at each length appear as constituents in correspondingly high frequency bundles of the next longer length. As is shown in Table 2, the lexical bundles at each length in Hawkes are almost double the length of those in Yangs. The discrepancies can be attributed to a number of factors including but not limited to (1) the translators' language backgrounds, (2) the translation skopos, translation settings, characteristics of the presumed receivers, and (3) the social, political, and ideological milieu in which they lived and worked.

While exploring identities, Hawks used longer sentences than Yangs which is indicative of more prevalent bundle use in Hawkes than in Yangs. The disparity between the two versions can be accounted for by the translators' different language backgrounds. The Yangs collaborated when translating *Hongloumeng* with the husband orally translating the text and the wife typing the translation on a typewriter (Li et al., 2011, p. 163). This collaborative translation mode clearly relies more heavily on single words than formulaic language, which directly results in the underrepresentation of lexical bundles in Yangs when compared with Hawkes. On the contrary, David Hawkes was a retired university professor who translated *Hongloumeng* mainly out of his passion for Chinese literature and sincere fondness for the novel.

Next, with different readership in mind, it is not surpring that Hawkes and Yangs each used different stylistic devices. When Hawkes translated *Hongloumeng* upon the request of the Penguin publisher in the 1970s, his presumed receivers are English-speaking readers who by large lived in Anglophone countries. As noted in his remark, Hawkes worked his best for his readers to remember those unfamiliar names and follow the story flow with ease (Hawkes, 1979, p. 20). It is equally unsurprising that Hawkes used a larger number and variety of lexical bundles to recreate a fictional space which is, like in our real world conversations, full of speech acts, framings and involvement. On the contrary, Yangs was appointed with this important duty to present *Hongloumeng* which is heralded as a classic of Chinese literature to readers in and out of China. His readership is not bound with any country in particular, and many English or even bilingual speakers in China also read his work. Unlike Hawkes who struggled to ensure his readers find *Hongloumeng* an enjoyable read, Yangs needed not cater to specific readers, thus resulting in a translated text whose lexical bundles is limited in both number and variety.

Finally, The Yang couple were first delegated to translate *Hongloumeng* in 1961, but their translation activity was disrupted by a number of political movements, in particular the Cultural Revolution (1966–1976) (Yang, 2002, p. 215). It can be observed that the literal translation approach adopted by the Yangs served as a “shield” to protect against the latent political risks that might arise from an overinterpretation of the text. In fact, this self-censorship practice at the time has given rise to a number of Chinglish words and phrases such as “roadist” (a person who advocates and preaches a certain doctrine) and “running dogs” (lackey) as a result of literal translation from Chinese. In contrast, Hawkes was not constricted by a similarly turbulent and intense political environment. Thus, it can be argued that he had more room to accommodate the subtle nuances of the novel. This also helps to explain why the translation by Hawkes demonstrates wider stylistic variation with abundant and varied use of lexical bundles as compared with the Yangs translation which adopts a more literal approach that leads to a relative underuse of bundles.

## Conclusion

This study adopted a corpus-driven approach to investigate the use of lexical bundles in the two translations of the Chinese literary classic *Hongloumeng* as a feature of translator's style. The findings show that Hawkes exceeds Yangs in presenting a greater number and variety of bundles, thus giving rise to sharp stylistic differences between the two translations of *Hongloumeng*. The stylistic differences as reflected in the use of bundles can be traced back to the translators' language backgrounds, the translation skopos and the social, political and ideological milieu in which the translations were produced. Since lexical bundles are often seen as building blocks of language, bundle use in different translations of the same original text can provide a useful starting point for exploring the stylistic idiosyncrasies of different translators. It should be noted, however, that the features identified in the current study are limited to this particular text rather than the translation/writing styles of Hawkes and Yangs in general. More research into other translations or even writings of the same translators can be carried out to confirm whether the stylistic features identified in this study are representative of the translators's style. Nonetheless, the current study has demonstrated that corpus-driven statistical and functional analysis of lexical bundles serves as a useful entry point for studying translator's style, thus representing a different methodological route than the one available through traditional corpus methods which make use of parameters such as type-token ratio and sentence lengths. Methodologically speaking, corpus-driven analysis of lexical bundles provides an innovative way to study and identify the stylistic features of different translators.

## Data Availability Statement

Publicly available datasets were analyzed in this study. The data is available in the form of corpus.

## Author Contributions

MA contributed in the literature review and overall discussion of the study. KL supervised the project and did quantitative analysis, handing of corpora, and corpus tools of this paper. All authors contributed to the article and approved the submitted version.

## Conflict of Interest

The authors declare that the research was conducted in the absence of any commercial or financial relationships that could be construed as a potential conflict of interest.

## Footnotes

^1^Since the cut-off rate of the lexical bundles is set at five, the lexical bundles which appear <5 were not extracted. For this reason, a further search was conducted to probe into the representation of these bundles in the Yangs corpus through function of Wordsmith. The search results show that “in the midst of” does not occur and “in the course of” occurs only one time in Yangs corpus.

^2^Due to the setting of cut-off rate, lexical bundles appearing less than five times are not extracted by Wordsmith. Further analysis using the function shows that this bundle appears two times in Yangs.

^3^Further analysis using shows that the three word bundle “by the hand” appears two times in Yangs.

^4^Further analysis using shows that this bundle occurs three times in Yangs.
